# Anticancer activity and chemoprevention of xenobiotic organosulfurs in preclinical model systems

**DOI:** 10.7243/2052-6199-1-4

**Published:** 2013

**Authors:** Robert E. Click

**Affiliations:** Altick Associates, River Falls, WI, USA

**Keywords:** Xenobiotic organosulfurs, 2-mercaptoethanol, cancer, radiation, viral, chemical carcinogen, spontaneous, garlic, dietary

## Abstract

There seems to be little doubt that xenobiotic and plant derived organosulfur compounds have enormous benefits for in vitro cellular functions and for a multitude of diseases, including cancer. Since there are numerous reviews on anticancer activities of plant organosulfurs, the focus herein will be on alterations associated with xenobiotic organosulfurs. Benefits of 2-mercaptoethanol (2-Me), N-Acetyl-cysteine, cysteamine, thioproline, piroxicam, disulfiram, amifostine, sulindac, celecoxib, oltipraz and their derivates on transplanted homologous tumors and on autochthonous cancers with a viral-, radiation-, chemical carcinogen-, and undefined-etiology are assessed. Because all organosulfurs were not tested for activity in each of the etiology categories, comparative evaluations are restricted. In general, all ‘appeared’ to lower the incidence of cancer irrespective of etiology; however, since most of these values were determined at ages much younger than at a natural-end-of-life-age, differences most likely, instead, reflect a delayed initiation and/or a slowed progression of tumorigenesis. The poorest, long-term benefits of early intervention protocols occurred for viral- and chemical carcinogen-induced cancers. In addition, once tumorigenesis was beyond the initiation stage, outcomes of organosulfur therapies were extremely poor, indicating that they will not be of significant value as stand alone treatments. More importantly, except for the lifetime prevention of spontaneous and radiation-induced mammary tumors by daily dietary 2-Me, similar life long prevention of tumorigenesis was not achieved with other xenobiotics or any of nature’s plant organosulfurs. These results raise an interesting question: Is the variability in incidence found for different organosulfurs associated with (a) their structure, (b) the length of the untreated latency period, (c) treatment duration/dose, and/or (d) the etiology-inducing agent?

## Introduction

There seems to be little doubt that xenobiotic and plant-derived organosulfur compounds alter many biological processes that translate into enormous benefits for a multitude of diseases. Whereas the majority of investigations on food derived organosulfurs focused on anticarcinogenic bioactivity, research with xenobiotic organosulfurs focused on enhancement of *in vitro* immune functions. Surprisingly, even with the knowledge that immune functions play a major role in controlling cancer, there seems to be little cross collaboration or acknowledgment by the groups studying these different sources. This is especially disconcerting since in many respects the structural requirements for biological benefits of many of the food sulfur compounds/ derivatives appear to be similar to, if not the same as, those postulated for bioactivity of xenobiotics [1–5].

Although there were a few early sporadic reports on organosulfur antioxidant benefits for virally induced and transplanted cancers [6–9], extensive investigations on the alteration of cellular events by organosulfurs began some 40 years ago when *in vitro*, cell mediated and humoral murine immune responses were shown to be dramatically enhanced by any of a multitude of structurally unrelated xenobiotic sulfhydryl compounds--2-mercaptoethanol (2-Me), dithiothreitol, reduced glutathione, and L-cysteine. Of these, the most effective was 2-Me, irrespective of whether it was added to protein-free or to autologous- or heterologous-sera supplemented culture media [10–14]. These findings led to an onslaught of reports defining benefits on immunological processes, and not surprisingly, on many other cell-types and processes (>1000 in PubMed). Regrettably, in many of these publications, the literature cited [15,16] as the origin of 2-Me’s dramatic enhancement is totally incorrect. The cited reports replicated and confirmed our original research presented in 1971 at the First Congress of Immunology (workshop #71) in Washington, DC [17], findings that were at the time in press, and were published in early 1972 [10,11].

As might be expected from the extensive literature generated from the *in vitro* findings, investigations on 2-Me administrated directly to animals soon followed (the 1980s). Makinodan and colleagues, the first to report on such benefits, demonstrated that the aged-associated decline in immune responsiveness both *in vitro* and *in situ* were corrected by either culturing with 2-Me or by a few (single or <5) injections of 2-Me [18–20]. A similar reversal of the age-decline of immune function in rats was subsequently reported [21,22]. Later, daily dietary exposure to 2-Me initiated at 16 weeks of age was shown to extend longevity [23], prevent both the decline of age-dependent, humoral and cell-mediated immune activity, curtail other aging processes associated with free-radical damage, and delay appearance of spontaneous liver tumors [23,24]; the cancer findings supported earlier results obtained with a different xenobiotic organosulfur, cysteamine [25–27]. Soon thereafter, a colleague, Lee Wattenberg and his collaborators reported that the potent dithiolthione, antihelmintic sulfur drug, oltipraz, inhibited chemical carcinogen-induced neoplasia in mice [28]. Within a year, multiple compounds present in cruciferous vegetables, which previously had been demonstrated to inhibit chemical carcinogenesis, were identified to be organosulfurs, albeit with many different structures [29–35]. This in turn resulted in very active investigations (the 1990s) on the potential for controlling cancer by dietary plant organosulfurs. Interestingly, upon reflection on the sequence of progression over the past 40 years (history), it might be concluded that the enhancement that 2-Me imparted on *in vitro* immune functions initiated, directly or indirectly, an evolution of a new subject-area of research, namely bioactivity of organosulfurs on cellular and disease processes. Indeed, over the past decade, description of multitudes of other processes altered by xenobiotic, food, and complex organosulfur compounds has occurred---it seems there is no end to the discovery of new benefits. However, the present review will be limited to tumorigenic processes, with the focus on xenobiotics and long-term outcomes; benefits for other processes will be the subject of a later undertaking.

## Results and discussion

### Plant organosulfurs

Investigations on food organosulfurs and their selenium analogs suggested that they possess therapeutic value for multiple diseases; initially the most extensively studied was cancer [reviewed in 36,37]. Specifically, epidemiological data indicated that the incidence of stomach [38] and prostate cancer [39] was lower in populations that consumed large amounts of garlic. However, an evidence-based review [40] of the literature concluded that “only a remarkably few studies with generally small numbers of subjects were scientifically sound” and that only a “modest reduction in the risk of cancer was documented”. More convincing anti-cancer benefits were described with *in vitro* and rodent models for some of the organosulfurs or derivatives present in Brassica and Allium foods. The focus of the majority of these studies was on alterations of a variety of chemical carcinogen-induced specific tumorigenesis processes; *i.e.*, induction of phase 2 carcinogen-detoxifying enzymes, inhibition of phase 1 carcinogen-activating enzymes, cell cycle arrest, and apoptosis; the latter process being preferentially enhanced in cancer cells relative to normal cells [41]. Even though the degree of alteration of these processes correlated with tumor progression, survival was at most modestly extended, and indeed, cures or preventions were rarely, if ever, achieved [29–36,42–45]. This raises the question: Why the disparity? An answer may need to consider that few animal investigations were directed at alteration of normal anticancer surveillance processes (immune-mediated?); instead almost all used xenograft transplant models and chemical carcinogens. In many respects, it could be argued that neither are hardly representative of normal cancer events. These type investigations further assumed that: (a) cancer caused by exposure to large doses of chemical carcinogens is a relevant model for tumorigenesis in humans; and (b) plant organosulfurs (i) can be consumed in sufficient quantities to be effective, (ii) are converted to bioactive forms *in situ*, and (iii) are not influenced by one another. Based on these uncertainties, it seems reasonable to ask: What are the realistic expectations of long-term, nutritional organosulfurs as preventive interventions? Evidence in the follow sections supports the conclusion that xenobiotics will be a more potent alternative.

### Xenobiotic organosulfurs

**Figure 1:**
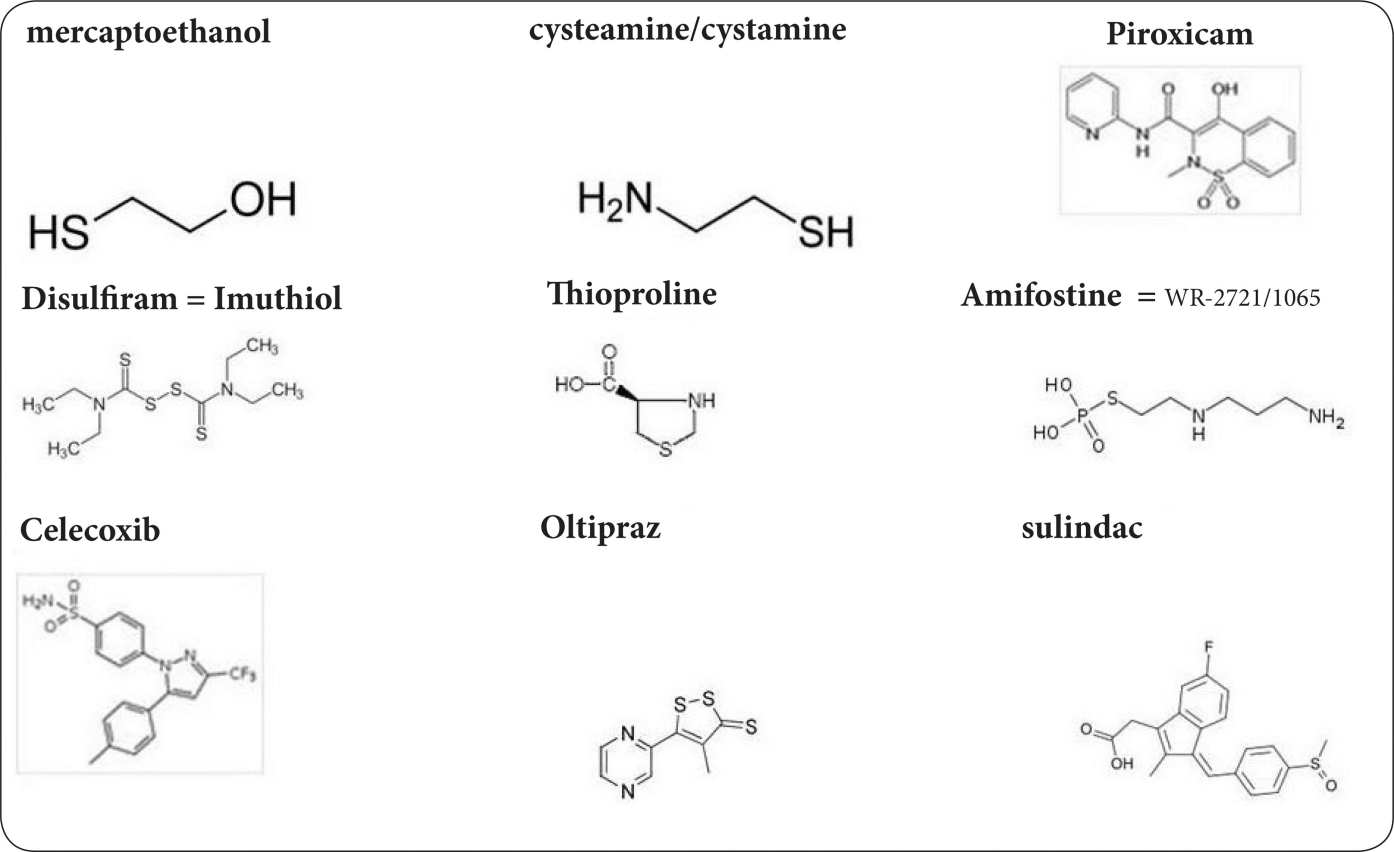
Structures of xenobiotic organosulfurs with anticancer activity.

The most extensive research with xenobiotic sulfur compounds originally focused on alterations of *in vitro* immunological processes; more recently benefits for a multitude of other cell-types, processes and diseases have been described [in preparation]. Since there is a lack of reviews on xenobiotic organosulfur bioactivities, the focus of this first report is limited to those that modify cancer processes (Figure 1). Each is assessed for alteration of transplanted autologous tumors and for autochthonous cancers with a viral-, radiation-, chemical carcinogen- or undefined-etiology (Table 1).

In many respects, bioactive organosulfurs from both sources share a number of similarities. Both are or can form ‘self’ or mixed disulfides, and those disulfides that are bioactive are structurally susceptible to enzymatic conversion to hypothesized H_2_S or sulfane sulfur [1–5,46–52]. And second, some of those not likely to form disulfide derivatives are still capable of generating H_2_S or sulfane sulfur. Arguments regarding these two potential end-products were recently reported [51,52]. However, other sulfur compounds (N-acetylcysteine, lipoic acid, glutathione, D-penicillamine, either as sulfhydryls or disulfides) have disease-altering capabilities, including cancer [53–57] and yet evidence that supports their generation of H_2_S or sulfane sulfur is lacking [1–5]. Irrespective of how xenobiotic or nature’s organosulfur compounds prevent development of, slow progression of, or are therapeutic post-occurrence of cancer (or any other disease), they have been categorized, directly or indirectly, as: (a) acting as free radical inhibitors/scavengers [58], (b) regulating gene expression [41,42,46,48–50], (c) maintaining critical allosteric disulfide configurations of cytoplasmic/membrane proteins [59,60], and/or (d) maintaining nature’s endogenous thiols— glutathione and thioredoxin—at an optimal redox balance for cellular functions [57,61,62]. Which, if any, of these categories best encompass clinical outcomes of structurally unique organosulfurs on different tumor-inducing insults remain to be defined; *in situ* mechanisms remain unresolved!

### Viral etiology

Murine strains infected with exogenous MMTV or endogenous MuLV [66] develop mammary tumors and leukemia respectively. Early investigations by Harman described the effects of dietary supplementation of four sulfur antioxidants, 2-Me, cysteamine, cystamine, and L-cysteine [6,25] with C3H/J virgin females and AKR/J males. Since these experiments were done prior to Jackson Labs deriving MMTV(S)-free C3H by foster nursing, mammary tumors were presumably induced by milk-borne virus transferred during nursing. Each of the four test compounds were incorporated into feed. One was fed once a day as a powder, which it was noted was the time the animals ate the most, and a second was fed as a pellet, which was supplied ad libitum—both test-diets were started at weaning. Obviously, these two methods of delivery would result in different and unequal intakes over a 24 hour period as well as possible alterations during pellet-formulation. The incidence of mammary tumors was not altered and there was no significant increase in longevity of C3H fed the powderdiet containing 0.5% or 1.0% cysteamine, cystamine, or cysteine; the slightly extended longevity found with 0.5% 2-Me (estimated at 20,000 ugm/day) was not statistically significant. In contrast, of the compounds tested in the pelletdiet, cysteamine at 1% (estimated intake of 40 mg/day) was the only one that increased median survival from 14.5 to 18.3 months (26%)—the importance of the diet formulation on survival by cysteamine was not addressed. In addition, this extension was accompanied by a delay in the onset and a lower incidence of tumors over a normal lifetime (how much was not indicated). In a similar viral model [64], 2-Me (daily intake of 2500–3500 ugm) added to water at weaning of C3H.OL and C3H.OH (H-2 congenic with C3H/HeDiSn) male and multiparous females did not change the 100% tumor incidence induced by exogenous MMTV(S), but did: (a) extend the age at which tumors became palpable by 31% (median); (b) increase median longevity 47%; and (c) increase median longevity, post-tumor detection, 75%.

With strains that develop leukemia, Harman found a 20 percent increase in the median survival for AKR/J fed (started at weaning) cysteine (0.5% and 1.0%), cysteamine (1.0%), or cystamine (0.5%) added to the powder-diet [6,25]. There was no alteration by the latter two at 0.5% and 1.0% or by 0.5% 2-Me. It was stated that prolongation was not due to prevention of leukemia, although a slowing would seem a probable explanation for the increased longevity. With the pelletdiet, median survival was increased 14.5% by cysteine only. Cystamine was tested at only 1.0% and based on the powder-diet results, would not have been expected to be effective at this dose. Interestingly, the non-sulfur, hydroxylamine in the pelleted-diet increased survival 8.3% at 1% and, 17.0% at 2%. With a different leukemic-prone strain, AKR/Cum, alteration of survival by 2-Me depended upon both dose and age at which treatment was initiated (Click, unpublished). At low doses (<400 ugm/day), longevity was shortened 13.7% and 15.4% from medians of 306 and 273 days for untreated males and females, respectively. In contrast, increase in longevity was directly associated with the age at which a high dose (≥3500 ugm/day) was initiated---the later the start-age (in utero, 35 days, 150 days), the more longevity was increased (8.6%, 22.8%, 36.3%). Leukemia status was not determined, but was likely curtailed by the most favorable 150 day start age; # surviving >365 days increased from 28.6% to 75% for males and from 12.5% to 50% for females. In summary, the impact of initiating organosulfur treatment at weaning on two naturally-occurring, viral-induced cancers was quite poor. In addition, effectiveness depended upon which drug was used, the tumor model, and the dose. The largest increase in longevity of the leukemia-prone strain by 2-Me was when treatment was initiated late in life, which will become apparent, is opposite that for tumors caused by other etiology agents.

### Radiation etiology

It has been known for many years that numerous factors influence radiation carcinogenesis in animals. Agents that enhance or suppress these processes were recently reviewed [65]. It is also known that many types of damage caused by radiation can be ameliorated by antioxidants [66], including some exotic botanicals [67]. Those containing sulfur are especially effective and will be the only ones considered herein.

Among radio-protective agents for humans, WR-2721 (amifostine) is one of the most commonly used [68,69]. This prodrug, after *in situ* dephosphorylation, is a potent sulfhydryl -containing protector against early and late radiation damage of most normal tissues. One of the earliest antitumor studies was undertaken with C3Hf/Kam mice that received a single localized gamma-ray dose of 34 to 57 Gy directed to a syngeneic methylcholanthrene-induced fibrosarcoma previously transplanted into one of the hind limbs [70]. Thirty minutes prior to radiation, ca. 12 mg WR-2721 was or was not injected intraperitoneally. Those cured of the transplanted tumor were then observed for up to 786 days post-irradiation for histologically-different tumors. Recurrence in both untreated and drug treated animals occurred around 300 days. Thereafter, the rate of development was slower in the treated group. At termination of the experiment, the incidence in treated mice was 26% and that in controls was 87%. Using an identical scheme of drug treatment, (C57BL/6 × BALB/c) F_1_ male and female mice were radiated (total body) with 10 cGy of neutrons [71]. Cancers of connective and epithelial tissue origins were identified at necropsy as animals succumbed from natural causes (mostly cancer). Surprisingly, the incidence and mortality due to spontaneous tumors in non-irradiated, WR-2721 treated and not treated, males or females was not significantly different. Secondly, radiation shortened the lifespan due to neoplasia-related deaths. Thirdly, the age of tumor-linked death was sex associated—for females, radiation-shortened longevity was altered only in the first half of their lives, whereas for males, alteration occurred only in the second half. In addition, there was no difference in the incidence of tumors in radiated vs. nonirradiated animals for either sex indicating that radiation simply shortened the latency. Because the shift was so minimal, the unanswered question is: was the latency of normal, spontaneously occurring cancer simply shortened or was a new cancer induced by radiation? Furthermore, the unaltered incidence of spontaneous arising tumors by a single WR-2721 injection raises the question as to its value; a question reinforced by the mere 65 day extended survival of WR-2721-injected animals that were radiated with a higher dose (206 cGy) compared to similarly irradiated, non-WR-2721 treated controls [72].

Anticancer activities of WR-2721 and cysteamine were compared in pregnant rats in which palpable mammary tumors were induced by sublethal irradiation [73]. All rats were implanted with the tumor promoter diethylstilbestrol a month after termination of nursing. No spontaneous tumors developed in those not irradiated, an obvious advantage for interpretations. Nontreated controls radiated with 1.5 or 2.6 Gy had tumor incidences at termination (one year of age) of 71.4% and 92.3%, respectively. A single injection of WR-2721 or cysteamine 30 minutes prior to the 1.5 Gy dose significantly lowered the incidence to 23.8% and 20.8%, respectively; a reduction that, in part, may have been a consequence of an extended latency. Tumor prevention by either drug was less effective at the higher radiation dose.

In a different series of investigations, the influence of cysteamine was compared to ranitidine on intestinal metaplasia induced by irradiation of male rats. At the age of 5 weeks, the animals were locally radiated in the gastric region with 10 Gy of X-rays at 3-day intervals for a total dose of 20 Gy [74]. After irradiation, the rats received either ranitidine (0.02% in their diet) or cysteamine (0.1%) in drinking water. Unfortunately, the start date and length of treatment given were not compatible with the age at which tumors were assessed—treatment was continued for 2 months after the animals were sacrificed! At 7 months, the incidence and number of metaplasia foci in those that were treated with cysteamine were significantly lower than that in nontreated controls, whereas in those given ranitidine, the incidence was higher than controls. Thus, two organosulfurs sharing the cysteamine backbone (HS-C-C-NH_2_ and R–C-S-C-C-NH-R’) resulted in opposite outcomes for radiation-induced tumor formation.

Nonsteroidal antiinflammatory agents also altered neoplasia induced by radiation. Wistar female rats treated orally with 8.0 mg/kg piroxicam 30 minutes prior and 24 hours after localized pelvic 2250 cGy were found to have a significantly decreased incidence of, as well as a delay in, endoscopically detectable colonic cancer (primarily adenocarcinomas). When the experiment was terminated, 15 of 19 (79%) animals not treated had cancer compared to only 8 of 20 (40%) treated. The first cancer detected in a control animal was at 15 weeks post-irradiation compared to 36 weeks for a treated animal [75], suggesting that the lower incidence at one year post-irradiation was most likely a consequence of a delayed/slowed progression. In a more complex model [76], male CBA mice were fed a diet that, in part, included two organosulfurs, lipoic acid and N-acetyl-cysteine as part of a multi-antioxidant formulation. This diet reduced the risk of developing 0.5 Gy iron ion or 3 Gy proton-induced malignant lymphoma (>20%) and rare tumors (>10%) to almost that which occurred spontaneously (7% and 2.5%) over two years. Although it is not possible to access the importance of individual components, daily treatment with lipoic acid alone retarded progression of a xenograft-implanted, SkBr3 breast cancer cell line, strongly implicating the importance of a compound that occurs naturally as a sulfhydryl or disulfide [56].

In a more recent study [77], exposure of long-lived, B10.A(4R) mice to sublethal, 5.5 Gy ionizing gamma-rays at 288 days of age resulted in a 43% incidence of palpable mammary tumors over a normal lifetime. This incidence differed significantly from the 0% in (a) 44 non-irradiated controls that were or were not exposed to 2-Me (10^−2^ M) drinking water (daily intake of 2500–3500 ugm) starting at 90 days of age—2-Me treatment was terminated in half of these 24 hours after being radiated whereas the others were continued on treatment for their remaining lifetime, and (b) 50 irradiated that were treated continuously with 2-Me irrespective of whether the start days were at 90 day of age or 24 hours post radiation. An unexpected result of these studies was that irradiation significantly (P = 0.0002) shortened longevity 29% of animals pretreated with 2-Me from undefined causes (there was no obvious signs indicative of cancer). This finding has relevance for the controversy on ’long term survival/safety’ of currently used antioxidants as free radical scavengers in humans receiving radiotherapy [78,79] and the very recent concerns regarding the increased cancer incidence in pediatric patients due to the increased use of computed tomography [80].

In summary, a single or <5 injections of WR-2721, cysteamine, or piroxicam protected against radiation damage, but did not prevent development or progression of cancer. Delayed/slowed development is the most likely explanation for the lower incidences found, since in most studies, cancer was not determined over an entire normal lifespan. There may be a difference in outcomes after total body versus localized radiation---cancers induced by the latter appear to be more amenable to prevention by a single injection. A reasonable conclusion is that limited injections are not a practice that will successfully prevent radiation-induced cancer. Lastly, continuous exposure of mice to 2-Me, either only prior to, only post, or both prior to and post, sublethal total body irradiation prevented development of mammary tumors for an entire lifetime; no effort was made to examine for other types of tumors, although there were no obvious indications of any. An unexpected finding however indicated that there should be some caution in designing radiation protective protocols, since pretreatment with 2-Me for many weeks prior to total body radiation created a radiation-sensitive process that was manifested later in life by a shortened longevity. This has special relevance regarding ‘long-term survival’ of radiated patients that are ‘protected’ with various antioxidants [78,79], many of which possess potential active/activatable sulfur. Safe use of antioxidants for radiation protection needs to consider the: timing of antioxidant exposure relative to irradiation; structure of the antioxidant (sulfur vs. nonsulfur); duration of treatment; and the fact that sulfur containing antioxidants impact biological processes by means other than as freeradical scavengers [1–5,46–52,81,82].

### Chemical carcinogen etiology

#### Disulfiram and other dithiocarbamates

Some of the earliest reports on organosulfur alterations of cancer were with disulfiram (200 mg/day), dimethyldithiocarbamate, and benzyl thiocyanate (cystine was ineffective). When added to the diet of 6 week old female Sprague-Dawley rats one week prior to oral intubation of 12 mg 7,12-dimethylbenz[a]-anthracene (DMBA), the 59–79% (three separate experiments) incidence of mammary tumors in untreated animals was reduced to 8–22%, 33%, and 8% by the three antioxidants at 23 weeks of age respectively; the number of tumors was also reduced. Further, a single, oral delivery of disulfiram at 24 hours before DMBA reduced the incidence from 59% to 11% [28]. In separate studies, disulfiram added to the diet (intake of 180 mg/day) for one week prior to and during carcinogen exposure, reduced the incidence of N-2-fluorenylacetamideinduced (2-FAA) mammary tumors by 50% and extended the mean latency period from 5 to 10 months; there was no effect on mammary tumors induced by the derivative, N-hydroxy-N-2-fluorenylacetamide (N-OH-2-FAA) [83].

Even though disulfiram was approved and used as a common treatment of alcoholism, after the late 70’s-early 80’s, interest as an anticancer drug waned. It resurfaced within 10–15 years as a means to control chemical carcinogenic-induced tumors in rodents. It: (a) completely prevented the occurrence of benzo[a]pyrene (BP) induced tumors in the fore-stomach of Ha/ICR female mice terminated at 29 weeks but did not alter induction of pulmonary adenoma formation in A/HeJ female mice at 31 weeks [28]; (b) completely prevented 1,2-dimethylhydrazine-(DMH-) induced tumors of the large intestine of CF1 mice at 36 weeks [84]; (c) reduced liver tumors (66% and 95%) induced in rats by either diethylnitrosamine (DENA) or dimethylnitrosamine (DMNA) [85]; (d) prevented liver tumors, had no effect on esophageal and urinary bladder tumors, and actually increased lung tumors from 0% to 30% in rats, all induced by N-nitrosodibutylamine (NDBA) [86], and (e) reduced urinary bladder cancer from 100% to 13% in rats given N-n-butyl-N(4-hydroxybuty)nitrosamine (BHBN) [87]. Surprisingly, when co-administrated with (a) DMNA, cell carcinomas of the paranasal sinus that were not found when either was given alone increased [88], and (b) NDBA, formation of lung tumors not normally induced by either individually were found [86]. It should be stressed that none of these treatments were followed for an entire lifetime. In summary, disulfiram effectively delayed and slowed progression of tumorigenesis in different organs induced by any of a number of different carcinogens. For other organs it was ineffective and when given in combination with certain carcinogens, actually enhanced tumorigenesis. It should not be unexpected that in several clinical trials, the results have been disappointing (www.clinicaltrials.gov).

Current postulated mechanisms of disulfiram activity appear to be multi-faceted with a complete understanding yet to be forthcoming. Based on investigations with cells in culture, various findings include: (a) cellular proteasome functions are inhibited [88,89], (b) inhibition of DNA methyltransferase (DNMT-1)--an enzyme that via a thiol group enhances global 5 methyl-cytosine content--resulted in a reduced level of methylated cytosine and a re-expression of epigenetically silenced genes that led to a reduced growth of prostate cancer cells *in vitro* and to a 40% reduction of growth as a xenograft [90]; (c) diethyldithiocarbamate (the active moiety) increased oxidative stress via lowering the level of reduced glutathione. This lower level was associated with DNA fragmentation and cell death [91], results that were reversed by pre-treatment with N-acetyl-cysteine [92–94]; and (d) increased mitochondrial antioxidant enzymes that were found to decline as tumor specific miRNAs declined [95,96]—miRNA levels that were returned to normal by disulfiram. Furthermore, these new levels were accompanied by lower levels of antioxidant proteins even though there was no decline in their messenger RNAs.

#### Cysteamine

Using the rat-DMBA mammary tumor model (15 mg/kg DMBA IV), cysteamine was tested for anticancer activity by IP injection at a dose of 150 mg/kg 20 min prior to and 5 and 24 hr after DMBA [26]. Tumor incidence at 4 months was 67% (12 of 18) for those receiving only DMBA and 26% (4 of 17) for those treated with cysteamine. The incidence increased with time in both groups and by 11 months the incidence and total number of tumors in the cysteamine-treated animals was the same as that in the 4 month, DMBA non-treated group. Thus, cysteamine delayed initiation and/or slowed progression, but did not prevent induction. In contrast to extended mammary tumor latency, induction of adrenal necrosis and lesions of the small intestinal epithelium was not altered [26].

In a separate study, the 80% rat-DMBA mammary tumor incidence was reduced to 50% by cysteamine and to 44% by the selenium disulfide analogue, selenocystamine [99]. In animals with tumors, there was no statistical difference in the total tumor yield (2.82 vs. 2.92/animal), even though the animals were treated for a much longer period; *i.e.*, the drugs were added to the diet 2 weeks prior to oral administration of DMBA at 8 weeks of age and continued for the entire 31–33 week duration of the experiment. Interestingly, the level of cysteamine required to obtain an inhibition comparable to that of the selenium disulfide analog was 500–750-fold higher. A more appropriate comparison of the analog would have been to cystamine (the sulfur disulfide of cysteamine) based on *in vitro* requirements [2] and *in situ* effects of cysteamine and cystamine [27].

Three other rat cancers in which cysteamine significantly reduced the incidence and number of tumors were gastric adenocarcinomas induced by N-methyl-N’-nitro-N-nitrosoguanidine (MNNG) [98], colon tumors induced by azoxymethane (AOM) [99], and heptocarcinomas induced by N-nitrosomorpholine (NNM) [100]. In each model, alterations were postulated to be mediated by catecholamines, specifically norepinephrine [101]. Again, as with most chemical carcinogenic agents, benefits for long-term survival were not determined—instead animals were sacrificed for biochemical and molecular analyses at pre-old-age stages.

#### Oltipraz and dithiolethione derivatives

Oltipraz, an antihelmintic (schistosomicide), and related dithiolethiones were found to possess anticancer activity. The primary metabolites of oltipraz generated by two major pathways common to various mammalian species are: “oxidative desulfuration of the thione, which does not seem to be metabolized further”; and second, “desulfuration, methylation, and cleavage of the dithiolethione ring disulfide bond, followed by cyclization of the resulting unstable intermediate into pyrrolopyrazines” [102], which can be metabolized to other nonfunctional oxidized forms. Some the earliest benefits of oltipraz were reported by Wattenberg and his collaborators [103]. They demonstrated that a single oral dose administered 24 or 48 hours prior to BP, also given orally, reduced the number of carcinogen-induced pulmonary adenomas and tumors of the forestomach in female ICR/ Ha mice. Formation of pulmonary adenomas induced by oral administration of diethylnitrosamine or uracil mustard carcinogens were also reduced by oltipraz when given orally 48 hours earlier, but not as pronounced as that found for BP. Others found it inhibited chemically induced carcinogenesis of bladder, colon, breast, stomach, and skin cancer models [104 and ref therein]. In studies with the urinary bladder-specific carcinogen N-nitrosobutyl(4-hydroxybutyl)amine (BBN), it effectively reduced the incidence of tumors in conventional C57BL/6 mice, but was ineffective in C57BL-*Nrf2*^−/−^ mice [105]. These results indicate that the nuclear factor, erythroid derived-E2-related factor 2 (*Nrf2*) pathway and its downstream target genes are responsible for BBN detoxification via phase 2 enzymes leading to diminished carcinogenesis.

Based upon induced hepatic phase II enzyme activities *in vitro* and a reduction in presumptive preneoplastic lesions in an aflatoxin B1-induced hepatic tumorigenesis rat model [106], various dithiolethione derivatives were compared for their ability to alter enzymes and disease. For these experiments treatment was started 3 weeks prior to a two week carcinogenic exposure and the experiment was terminated after another 5 weeks. Dietary concentrations of oltipraz and 17 derivatives induced hepatic phase II enzyme activities in vivo as well as produced marked inhibition of tumorigenesis [107]. Functional analysis indicated that gene alterations were associated with glutathione metabolism and with the *Nrf2* pathway. Of these compounds, 15 produced greater induction of NAD(P) H:quinone reductase and 11 yielded greater induction of glutathione S-transferase than oltipraz. Nine of these, spanning a range of gene alterations, were further tested for their ability to prevent AFB1-induced tumors. Six were found considerably more effective than oltipraz; interestingly, the most potent was the parent compound, 3H-1,2-dithiole-3-thione (D3T). An inverse correlation was found for in vivo phase II enzyme induction and tumor chemoprevention, indicating that enhancement of carcinogen detoxification pathways was a major contributor in preventing AFB1-induced cancer. Three of these compounds were further tested for their ability to induce specific gene functions. The parent compound, D3T, and oltipraz induced 226 common, differentially expressed liver genes 24 hours after Fischer F344 male rats were given three single doses on alternative days [108]. In contrast, forty genes had distinct responses; in addition, the response efficacy to oltipraz was weaker than that to D3T. Further studies demonstrated a comparable inactivation of protein tyrosine phosphatases by D3T and oltipraz via covalent modification of cysteine residues at the active site. This inactivation could contribute to the activation of *Nrf2* via the ERK1/2 signaling pathway [109]. In addition, oltipraz inhibited the enzyme, phosphatase SHP2, which may underlie its antiangiogenic properties. Disappointedly, similar differences in mean levels of rectal tissue and lymphocyte GSH and GST were not found during a 6 month, Phase I study with 26 patients that previously had undergone resected colon polyps or were first-degree female relatives of breast cancer patients [110].

#### Nonsteroidal anti-inflammatory Drugs (NSAIDs)

Another group of agents with anticancer properties, nonsteroidal anti-inflammatory drugs (NSAIDs), both non-sulfur and sulfur, are approved for treatment of diseases in humans, primarily for pain and inflammation. Oxicams (Piroxicam) and Sulindac are nonselective inhibitors (inhibit both COX 1 and 2), whereas Coxibs (Celecoxib) are sulfonamides with activity specific for COX-2. COX-2 specific inhibitors are preferred because of less serious side effects in the GI tract. Since benefits of various NSAIDs are reviewed elsewhere [111,112], only those in which their sulfur component impart benefits are considered herein.

#### Oxicams and coxibs

Male Sprague-Dawley rats fed a high dietary dose (130 to 195 ppm) of piroxicam had fewer methylazoxymethanol acetate (MAM)-, N-methylnitrosourea (MNU)-, or AOM-induced intestinal tumors [113–116]. In these studies piroxicam was fed 1 wk after initiation of or 1 wk before, during, and after carcinogen treatment. A significant reduction in the incidence of intestinal tumors and number of tumors/animal at 5 months occurred relative to that of controls. Similar results were found for AOM-induced colon tumors in F344 rats when any of four doses of piroxicam were initiated at either one or 13 wks post exposure to carcinogen. However, if started at 23 wk after carcinogen exposure, only the two high doses resulted in slightly lower incidences and number of adenomas/adenocarcinomas per tumor-bearing animal. Furthermore, piroxicam had no effect on the incidence of AOM-induced tumors of the duodenum, and had no consistent alteration of the incidence of ear duct tumors, primarily squamous cell carcinomas [115]. It was concluded based on the latter results, plus those described for male Sprague-Dawley rats exposed to MAMA [116], that “the spectrum of tumor types that are susceptible to piroxicam is not universal and it does not prevent or cure neoplasia”—it like most other sulfur chemicals simply delays initiation or slows progression. Similar reductions in the incidence of oral cancer in male F344 rats exposed to water-laced 4-Nitroquinoline 1-oxide (NQO) and breast cancer in female Sprague–Dawley rats gavaged with DMBA were found after piroxicam [117] or celecoxib [117–120] treatments. The experiments were terminated at 26 and 17 weeks respectively and prevention was not obtained. Just as found for miRNA alterations by disulfiram [95,96], downregulated miRNA (#29c), a tumor suppressor, was restored by celecoxib in human gastric cancer cells [121]. Disappointedly, analysis of two sets of data from the National Health Insurance Research Database lead to the conclusion that two selective COX-2 inhibitors, celecoxib and rofecoxib, may at most, benefit 10% of colorectal cancer patients [122].

Based on the inhibition of chemically induced colon tumors, plus the curtailment of Ornithine Decarboxylase (ODC), an enzyme involved in polyamine biosynthesis, in rodents by indomethacin suggested that lowering prostaglandin activity with specific inhibitors may further reduce tumorigenesis [123]. This was tested with D,L-α-difluoromethylornithine (DFMO), a specific irreversible inhibitor of ODC. Test diets were started 1 week prior to the first dose of AOM, and the rats were sacrificed 26 weeks later. Those that received either 0.05% or 0.1% DFMO in the drinking water or a high dose of piroxicam (2.6 mg) developed significantly fewer intestinal tumors than in controls. A low dose of piroxicam (1.3 mg) had no effect; however when combined with the low dose of DFMO, each acting through a different mechanism, reduced tumor formation more than DFMO alone [124]. In a separate report, certain dose combinations of these two drugs resulted in complete prevention of tumors induced by AOM for 56 weeks [125]. Similar benefits were obtained with a different COX-2 specific inhibitor, C-phycocyanin, in combination with piroxicam in DMH-induced precancerous colon polyps [126].

Based on the positive results obtained with rodents, piroxicam alone and in combination with either Deracoxib, cisplatin, Mitoxantrone, doxorubicin, or surgery was evaluated in dogs for anticancer benefits for urinary bladder, inflammatory mammary carcinoma, and oral squamous cell carcinoma [127 and ref therein]. Tumor-free survival, overall survival and biological response rates were at most modest. The combination of piroxicam and surgery appeared to result in the best survival advantage.

#### Sulindac

As a prodrug, it undergoes reversible oxidation/reduction to the sulfide metabolite, a potent inhibitor of prostaglandin (PG) production, or is irreversibly converted to the sulfone metabolite, which was originally described to lack pharmacological benefits [128]. Sulindac was effective at preventing intestinal tumors in familial adenomatosis polyposis patients that inherit a mutant allele of the *Apc3* gene [129,130], and in inhibiting tumor formation in a mouse model (Apc/Min) in which an allele of the homologous mouse *Apc* gene was inactivated by a mutation [131,132]. Similar inhibition was reported for (a) survival of malignant glioma cells *in vitro* due to a consequence of activation of an endoplasmic reticulum stress response (ERSR) [133]; (b) initiation of colon tumors induced in conventional mice by exposure to DMH (it did not cause regression of established tumors) [134]; and (c) DMH- and AOM-induced colonic tumors in rats, again more effectively at the initiation than the progression stage [135,136].

Although sulindac sulfone lacked prostaglandin synthetase inhibitory activity [128], it was found to have cancer chemopreventive activity for MNU-induced mammary [137] and for AOM-induced colon tumors [138,139], although effectiveness depended upon the time of administration (incidence was reduced most effectively when treatment was during the initiation and early post-initiation periods–only minimal alteration occurred during the promotion/progression stage [140]. Thus, both the sulfide and sulfone metabolic products of sulindac were most effective when given early; in utero was slightly more effective than if started at weaning [132] and mechanism of chemoprevention was, in part, independent of the prostaglandin pathway.

### Transplanted tumors

One of the initial studies [141] in which organosulfurs were tested for effects on transplanted tumors, found that a number of antioxidants, including cysteine and cysteamine were not effective at preventing growth of Ehrlich ascites. In retrospect, such a finding is what might be predicted since this tumor lacks tumor-specific transplantation antigens [142]. These experiments were followed by tests with DL-2-mercapto-3-hydroxypropanal [9], and a series of reports on antioxidant’s alterations of spontaneously arising tumors that have a viral etiology [6,25], (see preceding Virus section) and of transplanted solid tumors that were maintained by passage in the strain of origin (except Krebs-2 tumor) [27]. The four tumors in the study were: (a) Krebs-2 in Swiss mice, (b) methylcholanthrene-induced MC sarcoma in CS7BL/P mice, (c) sarcoma 1 in A/J mice, and (d) BAC/P, a spontaneous breast adenocarcinoma derived from C3H/HeJ. Different antioxidants were injected IP daily, starting either on the day of tumor inoculation, at 24 hours or 7 days post tumor transplant. Treatment was terminated on day 12 for C57BL/P, A/J, C3H/He and day 18 for Swiss since Krebs-2 tumors grew at a slower pace. Cysteamine and WR-2721 antioxidants merely slowed growth without achieving any tumor free A/J and C3H survivors. The best benefits were obtained with C57BL/P males, in which there were ‘numerous’ (the incidence was not given) tumor–free survivors at 5 months for the two treatments started within 24 hours of tumor transplant. A lower incidence was found for Swiss mice transplanted with Krebs-2 tumors. In addition to differences between the various tumor/strain models, all antioxidants were not equally therapeutic. This was investigated more extensively with the C57BL/P MC-tumor model. Cysteamine was effective in its native form, but was ineffective when either the amino or thiol group, or both, were blocked. Mercaptopropylamine, the 3-carbon homolog of cysteamine, was not as effective as cysteamine. Thioglycerol, one of the most potent *in vitro* enhancers of immune functions [143], had only negligible activity. Cystamine, the disulfide dimer of cysteamine was completely ineffective----a result just opposite that found for enhancement of proliferation of sulfur-dependent L1210 lymphoma cells in the presence of a required, exogenous source of diamine oxidase [2]. The most surprising result was that during a12 day treatment with the disulfides of 2-Me or cysteine, tumor growth was enhanced 214 or 187%!!!!!! In a different tumor transplant model, NAC was found to be ineffective, unless combined with an adoptive IL-2/LAK cell protocol against transplanted UV radiation-induced fibrosarcoma in C3H/HeN mice [144].

In assessing potential benefits and mechanisms of cancermodulating agents for treatment of human cancer, ideal model systems would utilize ‘normal’ immunocompetent animals because autochthonous organ-related tumors are: (a) located in ‘normal’ anatomical sites; (b) characterized by cellular heterogeneity; and (c) more representative of human disease counterparts. Thus, even though this section on autochthonous transplanted tumors is included, investigations relating to xenograft-transplanted tumors will not be considered. Results obtained with this model have been extensively reported by others and are almost exclusively related to chemical carcinogenic processes, plus being an unnatural model, have yet to contribute information, outside carcinogenic molecular processes, pertinent to cancer prevention/cures.

### Spontaneous (undefined etiology)

Sulfur antioxidants reported to have benefits for cancer of undefined etiology include (WR-2721), N-acetyl-cysteine (NAC), Thioproline, sulindac, and 2-Me. In early studies with WR-2721, a single injection of 400 ug/gm body wt did not alter spontaneous tumor development in (C57BL/6×BALB/c) F_1_ mice even though there was a slightly extended latency of tumor development post radiation--discussed above [71,72]. Investigations with NAC were mainly focused on C57BL/6-*Atm*^−/−^ (AT-mutated deficient) mice, a model of the human disorder ataxia telangiectasia (AT), in which both have abnormal humoral and cellular immune functions [145]. Treatment with NAC increased median survival of this strain from 50 to 68 weeks and reduced the incidence of thymic lymphoma two-fold [146], presumably due to a lowering of the enhanced oxidative stress created by an increase in reactive oxygen species (ROS) or to an abnormal response to ROS. This conclusion is based on a similar ROS alteration by the unrelated antioxidant Tempol, which doubled the lifespan of this strain by delaying the onset of thymic lymphoma [147].

In humans, reflux disease is associated with Barrett’s esophagus which may lead to an increased risk for esophageal adenocarcinoma (EAC), in part due to reactive nitrogen species. Thioproline (TOP) was tested in a Wistar rat model of human duodeno-gastroesophageal reflux disease that was created by anastomosing the jejunum side-by-side to the esophagogastric junction which allowed retention of normal stomach function [148]. Treatment with this nitritetrapping scavenger was started after surgery and continued for the duration of the experiment (70 weeks post op) by supplementation in the feed at 0.5%. Interestingly, it did not suppress the overexpression of inducible nitric oxide synthase (iNOS) and did not significantly alter the rare occurrence of esophageal squamous cell carcinoma (5.6% in controls and 7.7% in TOP treated). The mechanism suggested to explain the absence of EACs in TOP treated animals versus the 38.9% incidence in the control group was: TOP “inhibits not only the production of nitroso compounds by nitrite-reducing bacteria but also reactive nitrogen species (RNS) such as nitric oxide (NO), peroxynitrite (ONOO^−^) and N-nitroso compounds derived from reflux of duodenal contents”.

Another organosulfur investigated was sulindac and its two bioactive metabolites in the C57BL/6J-*Min*/+ (Min-mice) model of familial adenomatous polyposis. In the first study, sulindac resulted in both a reduced incidence (100% to 10%) and number of tumors per mouse (11.9 vs. 0.1) when assayed 70 days post treatment at 110 days of age [149]. In an essentially identical protocol, the sulfide derivative also [150] resulted in a reduced incidence (100% to 30%) and number of tumors per mouse (33.2 vs. 0.6). These reductions were accompanied by decreased levels of PGE2 and increased enterocyte apoptosis. These results contrast to the ineffectiveness found for the sulfone derivative (100% incidence and nonsignificant lowering of tumors/mouse to 21.9 [150]. This failure differed from its positive alteration of carcinogen-induced rat mammary and intestinal tumors [137–139]. However, interpretations need to be made with caution because ineffectiveness occurred with a dose 2–10× lower (sub-threshold?) than that used for the positive benefits. Irrespective, the anti-tumor effects in Apc-deficient animals imparted by low doses of sulindac are mediated by the sulfide metabolite and the benefits correlate with suppression of prostaglandin synthesis.

And lastly, 3 independent laboratories described alteration of spontaneous occurring cancers via undefined etiology agents by daily continuous exposure to 2-Me [23,24,64,151]. In the first report [23], 16 week old (C57BL × C3H/Anf) F_1_ male mice were fed a diet either free of or containing 0.25% (w/w) 2-Me (this calculates to a daily consumption of ca. 7,500 ugm or ≤200 ugm/gm body wt) for the remainder of their lives. The median survival time was significantly increased from 840 to 938 days (an increase of 11.7%), in part due to a slower development of tumors (primarily hepatomas) The average incidence calculated from 4–5 different ages (all after the peak-incidence ages of 424 and 665 days for nontreated and treated respectively) was reduced from 84% to 60% by 2-Me. Unfortunately, examination for the presence of tumors was terminated at 134 weeks, namely at essentially the median survival age. Other changes that occurred that may have a bearing on tumor development was 2-Me delayed the decline of immune functions and slowed the appearance of free radical induced intracellular lipid peroxidation products. It is important to note that neither of these latter processes were prevented, they were simply delayed. The second study, with a different strain of mice, CBA/Ca also found that diseases associated with old-age were slowed [24,151]. The mice consumed considerably less (8 ugm daily) from their water starting at 5 months of age and continued until they were terminated at 20 months (610 days). The incidence of liver carcinomas was reduced from 45% to 6% and Dunn sarcomas from 8 to 3%. Other changes that occurred were an increase in humoral functions to heterologous sheep RBC antigens and a decrease in the autoreactive humoral response to homologous murine RBCs. In the most recent report [64] 2-Me completely prevented the 100% incidence of mammary tumors in untreated BXSB-*Yaa*^+^/J mice, which increased the median longevity to 954 days from 650. Initiation of treatment was begun at weaning (40 days) and continued for the entire lifetime by adding 2-Me to the drinking water. The average daily consumption ranged from 2800 to 3500 ugm or 60–80 ugm/gm body wt [57].

Since the only complete life-long prevention of cancer was achieved with 2-Me, it is intriguing to speculate on mechanisms and future benefits it might impart, IF the ‘poison’ stigma can be overcome. Realization of a treatment that positively alters (derails) any aspect of cancer will depend on (a) if it alters tumorigenic processes, (b) how to integrate it with endogenous (preventive) mechanisms of amelioration, and/ or (c) how to best incorporate it as an adjuvant with other interventions, especially ex vivo preparation of dendritic cell (DC), TSTA specific CTL, or anti-CD1 LAK vaccines. Such an effort seems warranted based on mechanism that can be envisaged from reports that demonstrated:

- 2-Me is the most potent enhancer of lymphoid functions of all species both *in situ* and in cultures supplemented with autologous sera,
- 2-Me inactivates functions of rodent and human Treg cells (cells that are a major impediment to *in situ* anticancer immune functions [152–155],
- thiols enhance CD4, CD8, and LAK proliferation and functions, all thought to play significant roles in cancer control [59,62,156–161],
- NAC, a relatively weak organosulfur, enhanced IL-2/LAK adoptive anticancer therapy of transplanted autologous cancer--some mice were cured [144], and
- age-associated depressed functions of DCs (and T cells) were partially restored by the sulfur drug, pyrrolidine dithiocarbamate [162].

## Conclusions

Whether any of the many structurally unique organosulfur drugs discussed herein as well as those present in plant-foods alter tumorigenesis by similar or distinct mechanisms is a difficult question that remains to be answered? Much of the uncertainty is because there is not a single model system in which each organosulfur was tested for anticancer activity. Thus, even though comparisons are severely restricted, a few generalized comments can be made. First, all organosulfurs ‘appear ‘ to lower the incidence of cancer; however, these values were invariably determined at specified ages much younger than a ‘natural, end-of-life-age’. Thus, it is more accurate to view them as a consequence of tumor initiation being delayed and/or progression being slowed. Other generalizations are

- reduced and oxidized forms (cysteamine/cystamine) may result in different anticancer benefits—results that are not always consistent with *in vitro* activities [1,2],
- continuous vs. single exposure, as well as initiation of treatment prior to or soon after the inducing-event, resulted in the longest prolongation of latency,
- anticancer benefits were not the same for all etiology agents, and in some cases benefits were even organ specific,
- most drugs were effective against any of a number of different chemical carcinogens,
- combination with other drugs may have an enhanced or detrimental anticancer benefit,
- essentially all were not very effective against the two tumor inducing insults, chemical carcinogens and viruses, and
- slowing or preventing initiation was more easily achieved than altering established tumors,

Based on these generalizations it is almost certain that many of the anticancer benefits of xenobiotic organosulfurs are by multiple mechanisms, just as was postulated for the many uniquely structured food organosulfurs [163]. The most recent hypothesis involve gene control via alteration of specific miRNAs; supportive evidence is the differential gene activation by different structured organosulfurs [107–109,164].

Finally, perhaps the most important conclusion from all the models summarized herein is that organosulfurs will likely not be of value as stand alone cancer treatments, but may have value if combined with other therapies. The different degrees of delayed/slowed progression vs. complete prevention do raise two important points. First, it reinforces the concept that prevention is far more achievable than curative treatments. And second: Is the variability in incidence found for different organosulfurs associated with (a) their structure, (b) the length of the untreated latency period, (c) treatment duration/dose, and/or (d) the etiology-inducing agent? An answer should be extremely valuable in defining organosulfur cancer modalities that will yield better outcomes than those presently being found in clinical trials (www.clinicaltrials.gov).

## Figures and Tables

**Table 1:** Benefits of individual organosulfurs--etiology agents, tumor types, and references.

Etiologic agent	Sulfur compound	Tumor	Animal strain	Benefit^1^	Reference
**Viral**	2-Me	mammary	C3H/J, C3H.OL	N & S/DP	6, 64
	2-Me	leukemia	AKR/J	N	6
	2-Me	leukemia	AKR/Cum	S/DP	Click, unpublished
	cysteamine	mammary	C3H/J	LP	25
	cysteamine	leukemia	AKR/J	S/DP	6
	cystamine	mammary	C3H/J	N	6, 25
	cystamine	leukemia	AKR/J	S/DP	6,
	L-cysteine	mammary	C3H/J	N	6, 25
	L-cysteine	leukemia	AKR/J	S/DP	6, 25
**Radiation**	2-Me	mammary	B10.A(4R)	LP	77
	amifostine	sarcoma	C3Hf/Kam	S/DP	70
	amifostine	mammary	rat	S/DP	73
	amifostine	multiple	(C57×BALB/c)F1	S/DP	71, 72
	cysteamine	mammary	rat	S/DP	73
	cysteamine	intestinal	rat	S/DP	74
	piroxicam	colon	rat	S/DP	75
	Combination NAC + LA	lymphoma & rare	CBA/Ca	S/DP	76
**Chemical carcinogen**	cysteamine	mammary	rat	S/DP	26, 97
	cysteamine	gastric	rat	S/DP	98
	cysteamine	colon	rat	S/DP	99
	cysteamine	liver	rat	S/DP	100
	disulfiram	mammary	rat	N & S/DP	28, 83
	disulfiram	forestomach	Ha/lCR	NLP	28
	disulfiram	lung	A/HeJ	N	28
	disulfiram	intestinal	CF1	NLP	84
	disulfiram	liver	rat	S/DP	85, 86
	disulfiram	bladder	rat	N & S/DP	86, 87
	disulfiram	esophageal	rat	N	86
	oltipraz	pulmonary & forestomach	ICR/Ha	S/DP	103
	oltipraz	bladder	C57BL	S/DP	105
	oltipraz	liver	rat	S/DP	106, 107
	oltipraz	colon, breast, stomach, skin	rat	S/DP	104
	piroxicam	intestinal	rat	N & S/DP	113–116
	piroxicam	many	canine	S/DP	127
	celecoxib	intestinal, mammary	rat	N & S/DP	117–120
	sulindac	colon	human	S/DP	129, 130
	sulindac	intestinal	C57min	PP	131, 132
	sulindac	colon	Swiss	S/DP	134
	sulindac	colon	rat	S/DP	135, 136, 138–140
	sulindac	mammary	rat	S/DP	137
**Transplant**	cysteamine	MC sarcoma	C57BL/P	S/DP & LP	27
	SH-blocked cysteamine	MC sarcoma	C57BL/P	N	27
	NH blocked cysteamine	MC sarcoma	C57BL/P	N	27
	cystamine	MC sarcoma	C57BL/P	N	27
	amifostine	MC sarcoma	C57BL/P	S/DP	27
	cysteamine	BAC/P	C3H/HeJ	S/DP	27
	cysteamine	sarcoma 1	A/J	S/DP	27
	cysteamine	Krebs-2	Swiss	S/DP & LP	27
	cysteamine	Ehrlich	Any strain	N	141
	2-Me disullfide	MC sarcoma	C57BL/P	enhanced	27
	thioglycerol	BAC/P	C3H/HeJ	N	27
	amifostine	BAC/P	C3H/HeJ	S/DP	27
	amifostine	sarcoma 1	A/J	S/DP	27
	amifostine	Krebs-2	Swiss	S/DP	27
	NAC	fibrosacoma	C3H/He	N	55, 144
**Spontaneous**	2-Me	mammary	BXSB-*Yaa*+	LP	64
	2-Me	liver	(C57×C3H)F1	S/DP	23
	2-Me	liver/Dunn	CBA/Ca	S/DP	24, 151
	amifostine	multiple	(C57×BALB/c)F1	N	71, 72
	NAC	T-cell lymphoma	129Sv/C57B6Atm-	S/DP	146
	thioproline	esophageal	rat	S/DP	148
	sulindac	intestinal	C57min	NLP	149, 150

1 N = none, S/DP = slowed/delayed progression, PP = <100% incidence (partial prevention), LP = lifetime, 100% prevention, NLP = non-lifetime, 100% prevention.
